# Contribution of Non-immune Cells to Activation and Modulation of the Intestinal Inflammation

**DOI:** 10.3389/fimmu.2019.00647

**Published:** 2019-04-10

**Authors:** Renata Curciarello, Karina Eva Canziani, Guillermo Horacio Docena, Cecilia Isabel Muglia

**Affiliations:** ^1^Instituto de Estudios Inmunológicos y Fisiopatológicos, CONICET, Facultad de Ciencias Exactas, Universidad Nacional de La Plata, La Plata, Argentina; ^2^Departamento de Ciencias Biológicas, Facultad de Ciencias Exactas, Universidad Nacional de La Plata, La Plata, Argentina

**Keywords:** gut inflammation, inflammatory bowel disease, intestinal epithelial cells, intestinal fibroblasts, immune cell activation/modulation, intestinal microbiota

## Abstract

The mucosal immune system constitutes a physical and dynamic barrier against foreign antigens and pathogens and exerts control mechanisms to maintain intestinal tolerance to the microbiota and food antigens. Chronic alterations of the intestinal homeostasis predispose to inflammatory diseases of the gastrointestinal tract, such as Inflammatory Bowel Diseases (IBD). There is growing evidence that the frequency and severity of these diseases are increasing worldwide, which may be probably due to changes in environmental factors. Several stromal and immune cells are involved in this delicate equilibrium that dictates homeostasis. In this review we aimed to summarize the role of epithelial cells and fibroblasts in the induction of mucosal inflammation in the context of IBD. It has been extensively described that environmental factors are key players in this process, and the microbiome of the gastrointestinal tract is currently being intensively investigated due to its profound impact the immune response. Recent findings have demonstrated the interplay between dietary and environmental components, the gut microbiome, and immune cells. “Western” dietary patterns, such as high caloric diets, and pollution can induce alterations in the gut microbiome that in turn affect the intestinal and systemic homeostasis. Here we summarize current knowledge on the influence of dietary components and air particulate matters on gut microbiome composition, and the impact on stromal and immune cells, with a particular focus on promoting local inflammation.

## Gut Inflammation

Inflammation is a central component of innate immunity, comprising the physiopathological response to infection or tissue damage. As a local response to cellular injury, it is initiated when tissue-resident cells of the innate immune system detect the damaging insult and alarm resident cells and circulating neutrophils. These cells migrate to the inflamed tissue, promote recruitment of inflammatory monocytes and potentiate the pro-inflammatory environment, allowing to deal with the harmful agent (1). Hence, the acute inflammatory response is marked by increased blood flow, capillary dilatation, leukocyte infiltration, and the localized production of chemical mediators, which serves to initiate the elimination of toxic agents and the repair of the damaged tissue. Hence, the acute inflammatory response is a physiological process committed to control an offending stimulus.

The intestinal mucosa has evolved as a well-structured barrier against physical, chemical, and microbial insults. The epithelial layer, mucus, antimicrobial peptides, secreted immunoglobulin A, and innate and adaptive immune cells, together help to establish a beneficial environment to tolerate the diverse community of microbes of the microbiota and food antigens. The intestinal mucosal surface constitutes the major interface between the internal tissues and a potentially hostile outer environment. To deal with this universe of antigenic components, intestinal homeostasis has been evolutionary developed through a constant crosstalk between metabolites and microbes of the microbiota, intestinal stromal cells, and the mucosal immune system. Perturbations of the homeostatic state can result in severe inflammatory conditions in the gut that may lead to tissue damage. Therefore, intestinal inflammation is a double-edge sword that should be tightly regulated. Although it is an essential component for immunosurveillance and host defense, chronic inflammatory processes may promote pathology such as inflammatory bowel diseases (IBD) (2), irritable bowel syndrome (IBS) (3), diverticular disease (4), food allergy (5), celiac disease (4), etc.

**Figure 1:**
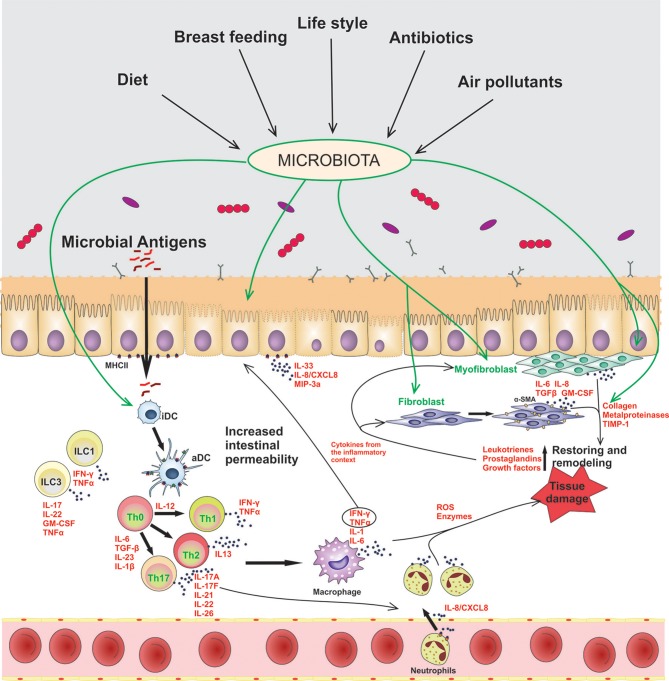
Contribution of epithelial cells and intestinal fibroblasts to the induction and modulation of mucosal inflammation in IBD. In this disorder the intestinal barrier permeability is impaired, allowing the passage of luminal antigens into the lamina propria. The exposure of stromal and immune cells to the luminal content induces cell activation, inflammatory soluble-mediators release (IFN-γ, TNF-α, IL-1β, IL-6, IL-17A), cell-crosstalk (represented with arrows) and neutrophil recruitment to the inflamed tissue. Several environmental factors can modulate the microbiota composition and the activation of stromal and immune cells in the gut. iDC, innactivated dendritic cells; aDC, activated dendritic cells; ILCs, innate lymphoid cells; Th, T helper cells.

In this homeostatic scenario, the controlled and physiological inflammation of the gut promotes a barrier permeability that allows the penetration of luminal antigens to the underlying mucosal tissue. In chronic inflammatory disorders, such as IBD, microbial components of the microbiota are translocated through the damaged mucosal barrier, and trigger and maintain a sustained inflammatory response, as it is represented in Figure 1. Epithelial cells, dendritic cells (DCs), macrophages, and innate lymphocytes (ILCs), which sense the presence of microbes or an altered tissue environment, are activated and promote the induction of the adaptive immune response. As it is summarized in Figure 1, the pro-inflammatory cytokines secreted by innate cells and activated T cells are key to amplify and perpetuate mucosal inflammation. Therefore, the mucosal immune system is responsible for the induction of the inflammatory process, while tissue damage results from continuous activation and differentiation of local cells, such as myofibroblasts, that release noxious mediators (2, 6).

## Non-immune Cells Involved in Gut Inflammation

### Epithelial Cells

The alteration of the intestinal epithelial barrier permeability leads to local inflammation. The fact that mice expressing dominant negative N-cadherin adherent junction protein suffer from spontaneous inflammatory bowel disease, clearly highlights the fundamental role of barrier integrity in the development of colitis and, probably, IBD (7). Many cytokines that are increased in IBD favor gut permeability, such as TNF-α. Indeed, impaired barrier function has been described for Crohn's disease (CD) and ulcerative colitis (UC) patients and it is a marker for predicting the course of these relapsing diseases (8, 9). Intestinal pro-inflammatory cytokines have been documented to modify the expression of different tight junction proteins. Claudins showed deregulation and differential expression in human active IBD (8, 9). However, studies in mice null for claudins and other tight junction proteins were controversial, demonstrating that other factors may contribute to gut permeability (10). Overall, these reported evidences highlight the relevance of epithelial cells in intestinal inflammation. Amongst these, Paneth cells secrete antimicrobial peptides that modulate the gut microbial composition (11), and it has been recently described that metabolites produced by luminal microbes control the secretion of these peptides (12). In other words, there is a mutual monitoring of the microbiota composition and the mucosal immune system. Defects in defensin production have been found in patients with CD and NOD-2 mutations (13). Taking into account the role of the microbiome in intestinal homeostasis, these findings should not be underestimated. Recently, a study involving Paneth cell deficient mice showed that they develop dysbiosis and visceral hypersensitivity (14). Other relevant epithelial cell type are Goblet cells, responsible for secretion of mucus and sampling of luminal antigens (15, 16). These cells showed a protective role in gut inflammation since MUC2-null mice developed spontaneous colitis (17), and patients with UC showed polymorphism in MUC2 in the Dutch population (18). Goblet cell loss and decreased mucous levels are commonly observed in UC patients, with endoplasmic reticulum stress and accumulation of MUC2 precursors (17, 19). In addition, micro-fold cells, or M cells, found in the follicle-associated epithelium of the gut, have a key function in the immunosurveillance of the luminal content (16, 20, 21). There is no report showing M cell dysfunction in colitis or any other inflammatory condition. Enteroendocrine cells, which are also sensors of gut luminal content, were found to be altered in mouse models of colitis and in patients with active IBD (22).

The most abundant cells in the epithelial compartment are the absorptive cells, which not only constitute a physical barrier against luminal antigens, but mediate the crosstalk between intestinal microbiome and the host immune system, mainly through innate immune receptors. Conserved molecular patterns are mainly recognized by Toll-like receptors (TLRs) and Nod-like receptors (NLR), among others, which are expressed along the intestinal tract (23–26). Healthy human small intestine expresses TLR-2 and−4 proteins, whereas high levels of TLR-5 are found in the colon. These receptors show a polarized distribution, being located in the basolateral membranes, ensuring that commensal bacteria do not trigger an inflammatory response in homeostasis (27). These particular expression patterns of innate receptors are modified in an inflammatory setting. IBD patients have increased levels of TLR4 expression, and lower level of TLR2 and TLR5 in epithelial cells, while TLR4 was shown to be expressed in the apical surface of epithelial cells (28). Current studies have demonstrated the importance of TLR1 in the prevention of gut inflammation (29). In addition to NOD-2 mutations, abnormal mucosal NLRP3 activity has been reported in IBD and in experimental colitis; GWAS studies revealed polymorphisms in these receptors (30–33).

Intestinal epithelial cells (IECs) also contribute to intestinal homeostasis through interaction with microbiota and secretion of TGF-β and IL-10 (34, 35). Although IECs crosstalk with T cells through cell-cell interactions (36), the role of these cells as antigen presenting cells *in vivo* is controversial (37). They express class 2 MHC, but no co-stimulatory molecules, though they do express the ICOS-L; of note, variants in this gene have been related to the IBD early onset clinical entity (38). Colonic epithelial cell isolated from active IBD patients have shown to secrete the neutrophil-attracting IL-8/CXCL8 (39, 40) and IL-33 (41–44), which contribute to inflammation (Figure 1). IL-1β, produced through activation of the inflammasome, and IL-17 are also secreted (45, 46), contributing to Th1 and Th17 responses in IBD. IL-21R expression is up-regulated in IECs of IBD patients, which leads to increased CCL20 synthesis, a T cell and DC chemo attractant (47, 48). IL-22, IL-31, and IL-33 have been also described to be augmented in IBD, which leads to deregulation of IEC proliferation and migration functions, whereas they stimulate IL-1, TNF-α, IL-6, and IL-8 secretion (49–51). Cytokines generated by innate and adaptive responses, on the other hand, interact with IECs, activating them and altering barrier permeability. It is well known that TNF-α induces IECs apoptosis (52) and that IFN-γ alters epithelial permeability by affecting tight junctions and bacterial translocation (53), as represented in Figure 1. All these factors contribute to immune cell activation and cytokine secretion which, in the context of IBD, favor an inflammation and damage perpetuation.

In conclusion, IECs are relevant cell sources of pro-inflammatory and regulatory mediators, which should be tightly controlled to achieve intestinal homeostasis.

### Fibroblasts

Intestinal myofibroblasts are localized beneath the epithelial compartment, particularly around the crypts of the small intestine and the colon, and participate in the repair process. Fibroblasts, which are involved in formation of the extracellular matrix (ECM), differentiate into contractile myofibroblasts, which are also involved in the inflammatory response to injury through the secretion of cytokines (54–56). Furthermore, the origin of myofibroblasts in chronic inflammation is not clear, and they may have an epithelial origin through an epithelial-mesenchymal transition (57).

Myofibroblast activation is associated with tissue injury and inflammation (Figure 1). They have the ability to migrate to the injured sites, where they contract the wound area and produce extracellular matrix components that restore and remodel the damaged mucosal tissue. However, persistent myofibroblast activation in an inflammatory environment may promote an irreversible damage of the affected tissue with fibrosis and cell proliferation in the submucosa (58). Cells can secrete a number of soluble cytokines (IL-6), chemokines (IL-8, MCP-1), growth factors (TGF-β, GM-CSF), collagen, metalloproteinases (MMP-1, MMP-3, MMP-9, MMP-12), and MMPs' inhibitor (TIMP-1), that attract other cells to perpetuate the inflammation. Depending on the balance of MMPs and TIMP-1, and collagen deposition, it will be the consequence of tissue remodeling in IBD, thus resulting in fibrosis, stricture formation or ulceration (59–62). It is unknown why in some cases the intestinal inflammation induces penetrating damage with perforation and fistulae, and with increased risk of colorectal cancer in UC patients, whereas in CD patients the long-term complications include abscesses, granuloma, strictures, obstruction, fibrosis, and stenosis. Overall, the control of myofibroblast differentiation is critical to prevent or reverse complications in CD and UC patients.

## Inflammation Is Modulated by Different Factors

It has been previously described that the immune system is regulated at different points for homeostasis, while aberrant or inappropriate regulation may result in failure to protect the body from pathogens or any injury.

Environmental factors influence the incidence and development of IBD in many ways that are not fully understood, with a higher incidence in developed countries and urban populations, compared to rural areas and underdeveloped regions of the world. It has been shown that young migrants from low incidence countries have a similar incidence of IBD compared to non-immigrants, highlighting the importance of the environment in these diseases (63). Several studies discuss associations of different dietary components in gut inflammation and IBD. High meat/fat (64) consumption has been linked to a higher risk of IBD onset, and studies in animal models correlate this finding with the high heme intake (65). Also, trans- and poly-unsaturated long-chain fatty acids have been related to the disease (66, 67), while unsaturated fatty acids seem to exert a preventive role (67). Emulsifiers, which are commonly found in processed foods, have a pro-inflammatory effect in the gut (68). On the other hand, dietary fiber, especially from fruits, has been associated to a lower IBD incidence (69). Also, vitamin consumption is thought to be beneficial (70). These results should now be reevaluated after the recently published study in which the effect of different dietary ingredients was addressed in the context of IBD, using experimental colitis models in pathogen-free and germ-free mice in order to identify specific triggers (71).

More recently, the microbiota has been targeted as a critical player in establishing and sustaining the tight equilibrium of the immune system that is constantly exposed to a myriad of antigens in the mucosal surfaces. The intestinal microbiota is key for maintaining intestinal homeostasis, and it may be involved in inflammatory disorders when the composition and diversity is modified, which is called dysbiosis. In all mucosa an associated microbiota has been described and it is known that the gut microbiota might be the most complex and dynamic one. It is established during the intrauterine life and modified after birth. Several factors have been described as contributing to condition the composition of the microbiota throughout life. There is a link between diet, gut composition and gut metabolism which undoubtedly impact on the gastrointestinal health. There is growing evidence that a dysbiotic intestinal microbiota is associated with immune and non-immune disorders. However, it is debatable whether dysbiosis is a cause or a consequence of the inflammatory process (72, 73). Commonly, dysbiosis implies a change from a diverse anaerobe community, rich in *Firmicutes* and *Bacteroidetes* to a lower diversity community with enrichment of facultative anaerobes including *Proteobacteria* and *Bacilli*, although it depends on the pathology (74). There is broad consensus that pathology-associated dysbiosis is accompanied with a restriction in the diversity of species (75).

Microbiota composition may be affected by different external factors: diet (fiber, calories, etc.), urbanization, use of antibiotics, age, mode of birth, exposure to air pollutants, etc. (76–78). A westernized diet with high red meat-high fat and processed carbohydrates content, is associated with a loss in gut microbial diversity, with an increase of pathogenic adherent-invasive *E. coli* (AIEC), as it has been reported in IBD patients (79). It has been demonstrated that a high-fat/high-sugar diet leads to dysbiosis with increased *Bacteroides* spp and *Ruminococcus torques* in mice (79). On the other hand, digestible fibers, which are fermented by bacteria in the gut, generate beneficial, anti-inflammatory short-chain fatty acids (SCFA). Modern diet components such as artificial sweeteners and emulsifiers are being subjected to investigation as they are suspected to induce dysbiosis (80–82). Human and murine studies have demonstrated that the use of antibiotics generates variations in gut microbiota mostly according to the type and the period of time used (78). Antibiotics directed to anaerobes, such as vancomicin, seem to have a more severe impact on gut microbiota composition (76). The contamination of food with particulate matter, as occurs in a contaminated environment, has shown to impair intestinal permeability and to generate inflammation, altering microbial gut composition (77, 83).

There are several evidences that highlight the relevance of the microbiota in IBD: germ-free animals do not develop experimental colitis (84), and antibiotic therapy has been successful in the treatment of CD, while it has given promising results in some forms of UC (84), etc. Dysbiosis has been well characterized in CD, with a decrease in *Clostridiales* and an increase in *Enterobacteriales* (85), while a diminished, but less defined, microbial diversity has been described for UC (85). Emerging insights on intestinal dysbiosis during immune and non-immune disorders has attracted the attention to target the microbiota composition as a novel therapeutic approach to control intestinal and extra-intestinal inflammation. However, it should be considered that the microbiota-derived metabolites are the true messengers that control the development, differentiation, and activity of the immune system associated to the local, and also, distant mucosa. Microbiota transfer strategies, or fecal microbiota transplant (FMT), have been used in Chinese ancestral medicine for centuries and have proven to be useful for *Clostridium difficile* recurrent infections (86). Several clinical trials have demonstrated that FMT restores gut microbial diversity, diminishing disbiosis and controlling mucosal inflammation. More recently, FMT has been explored in IBD (87). Most clinical trials and randomized controlled studies have been made in UC, with promising results when they were performed early in the disease course (88). Results in CD have been variable and rigorous randomized controlled trials are needed (89). More studies are mandatory to confirm the beneficial effect of this therapy.

Another therapeutic approach aimed to modify the gut microbiome has been the use of probiotics. Numerous studies using animal models (90–93) and *in vitro* approaches (94–96) provide evidence on the beneficial use of probiotics in colitis. Results depend on the specific strain of probiotics used, and vary with the experimental model used. In particular, *Bifidobacterium* and *Lactobacillus* are the most widely used probiotic bacteria (97), although strains of *E. coli* (98–101), *Propionibacterium* (102–105), *Bacillus* (106–108), and *Saccharomyces* (109, 110) are amongst those mostly studied.

Different mechanisms of action have been described for probiotics, including binding to IECs and thus preventing the binding of pathogenic microorganisms (109, 110), acidification of the lumen of the colon by nutrient fermentation, production of SCFA like acetate, propionate, and butyric acid as a source of energy, but also with immune modulating properties and anti-inflammatory effects (109, 110), as enhancement of epithelial barrier integrity (97), etc. Mack et al. (111) described an increased production of MUC2 and MUC3 secretion by epithelial cells stimulated with *Lactobacillus plantarum* and *Lactobacillus rhamnosus* in HT-29 intestinal cell line, while Anderson et al. described that *L. plantarum* MB452 increased the trans-epithelial electrical resistance of Caco-2 cells monolayer through the induction of tight junction proteins (112). Recently, components of *L. amylovorus* DSM 16698 cell wall demonstrated to have protective effects toward *E. coli* induced damage on Caco-2/TC7 cells, protecting membrane leakage and reducing the phosphorylation of the p65 component of the NF-κB intracellular signaling pathway (113). The inhibition of NF-κB activation has also been described for other *Lactobacilli* strains and for *Bifidobacterium* (114–116). The increased expression of tight junction proteins is linked to an activation of the ERK and p38 MAPK signaling cascades, via Toll like receptors (114). Probiotics have also been described for preventing IECs apoptosis by reducing oxidative stress, as in the case of *L. amylovorous* DSM16698 (117), and to inhibit apoptosis through the activation of the epidermal growth factor receptor by the release of a soluble protein known as p40 by *L. rhamnosus* GG (114, 118). In addition, probiotics act by diminishing pro-inflammatory responses and contributing to tolerogenic responses, modulating TLR-2 and TLR-4 signaling (114), and driving DCs to a suppressive phenotype, which further promote the generation of Tregs (119, 120).

It has been demonstrated in the inflamed gut, that the mucosal damage not only affects the epithelial compartment, but also colonic myofibroblasts located beneath the epithelium. It has been reported that the exposure to colonic microbiota products promotes cell activation through TLR (121). Beswick and col. demonstrated that isolated myofibroblasts from normal human colonic mucosa respond to TLR4 stimulation by LPS with the induction of PD-L1, which mediates the suppression of activated CD4^+^ PD-1^+^ T cell response and inhibition of IFN-γ secretion *in vitro*. Authors also showed that *in vivo* up-regulation of PD-L1 in colonic myofibroblast is MyD88-dependent in colitis (122). This provides evidence that colonic myofibroblasts might help to maintain the equilibrium between tolerance and immunity to protect the colonic mucosa against inflammatory responses toward the microbiota. In agreement with these findings, Scheibe and col. reported that IL-36R ligands released upon mucosal damage activate IL-36R^+^ colonic fibroblasts via MyD88, thereby inducing expression of chemokines, granulocyte-macrophage colony-stimulating factor and IL-6. These mediators induce the migration and recruitment of leukocytes and neutrophils to the inflamed colon and contribute to control mucosal healing (123).

On the other hand, dysbiosis and specific bacterial taxa correlated with fibrostenosis in a CD cohort study (32), although there are no studies showing the contribution of the microbiome to fibrosis and fibroblasts modulation in the gut. It has recently been published the first evidence that fibroblast activation and the intestinal fibrosis require specific microbial cues provided by the mouse microbiota. Authors reported that intestinal fibrosis is microbiota-dependent, by giving gavages of feces from germ-free mice, pathogen-free feces or healthy human donor feces, in TL1A-overexpressing mice. In addition, they found that the microbial composition affects fibroblast differentiation into myofibroblasts. Furthermore, they identified several candidates that correlated directly with the fibrosis degree in mice (123, 124). Although the key role of TL1A in inducing fibrostenosis is known (125, 126), the interplay between microbiota, TL1A and fibroblasts is novel (124).

Given that there are a number of chronic inflammatory disorders with fibrotic evolution, such as rheumatoid arthritis, cirrhosis, IBD, and pulmonary fibrosis, there is great effort to develop therapies that could control or reverse fibrosis. Particularly, intestinal fibrosis has been exclusively associated to IBD, mostly CD. The use of probiotics is becoming increasingly important for the prevention and treatment of gastrointestinal conditions. In a mouse model of DSS-induced colitis, administration of *Lactobacillus acidophilus* ameliorated collagen mucosal deposition and improved intestinal fibrosis. Indeed, *L. acidophilus* treated mice showed decreased α-SMA and collagen I expression levels, compared to untreated mice (127). Alternatively to probiotics, a role for Vitamin D in modulating the immune system and the integrity of intestinal epithelium and gut microbioma has also been proposed. In this sense, vitamin D supplementation improves IBD patient's condition (128) and regarding intestinal fibrosis, it was demonstrated that vitamin D exerted protective effects on colonic fibrosis caused by TNBS-induced chronic colitis, through direct inhibition of TGFβ-1/Smad3 pathway and up/regulation of vitamin D receptor in sub-epithelial myofibroblasts (129). More recently, the overexpression of fibroblasts activation protein (FAP), an inducible surface glycoprotein, has been associated with fibrosis in strictured CD patients. *In vitro* assays with anti-FAP treatment have shown promising results in controlling ECM deposition (130). Overall, despite the widely described contribution of fibroblasts to intestinal inflammation, the control of fibrosis in chronic intestinal inflammatory disorders still remains a big challenge for therapeutic purposes.

## Conclusions

As the intestinal mucosa surface constitutes the major surface of the body which is in direct contact with the outer environment, intestinal immune homeostasis must be accurately regulated. The interplay between commensal microbiota, intestinal stromal cells, and the mucosal immune system components should guarantee the intestinal homeostasis to avoid a sustained inflammation that could induce tissue damage. However, several factors can lead to inflammation through homeostasis breakdown. Figure 1 summarizes the main points that have been reviewed here. We have described what it is known so far about the role of epithelial cells and intestinal fibroblasts in the induction and modulation of mucosal inflammation in IBD. In this chronic inflammatory disorders the intestinal barrier permeability is compromised and the selective passage of luminal antigens into the lamina propria is altered, triggering cell activation, and inflammation. A plethora of evidences demonstrate the impact of dietary and environmental factors on the gut microbioma and on the modulation of the intestinal immunity. Notwithstanding the efforts made to find alternatives to conventional anti-inflammatory treatments (steroids, antibiotics, immunosuppressive drugs and biologics) by modulation of non-immune cells response, no current evidences arise that support the replacement of conventional therapies. Regarding probiotics, for instance, it has recently been demonstrated that the use of probiotics to achieve effective mucosal protection should be personalized according to the individual affection (131–133).

In conclusion, in this review we summarized the most recent findings in animal models and cohort studies, that show the contribution of epithelial cells and fibroblasts to gut inflammation with the influence of different environmental and dietary factors. Considering that the frequency and severity of IBD are increasing worldwide, changes on environmental factors and dietary habits should not be underestimated. Based on these observations, and those regarding the modulation of the intestinal microbioma and mucosal immune cells, it has been prompted to develop novel therapeutic interventions to prevent, control or reverse gut inflammation.

## Author Contributions

RC, CIM, KEC, and GHD conceptualized the review. RC and CIM provided an initial draft of the manuscript and KEC provided the figure. RC, CIM, and GHD performed the final editions.

### Conflict of Interest Statement

The authors declare that the research was conducted in the absence of any commercial or financial relationships that could be construed as a potential conflict of interest.
